# The Rosenberg European Academy of Ayurveda - Quality in Ayurvedic education in German-speaking Europe

**DOI:** 10.4103/0975-9476.74091

**Published:** 2010

**Authors:** Mark Rosenberg

**Affiliations:** *The European Academy for Ayurveda, Birstein, Germany*

## INTRODUCTION

### Ayurveda in Europe

As with all complementary systems of medicine, there has been an upsurge in public interest in Ayurveda. This is especially true in German-speaking areas of Europe, where alternative medicine is relatively popular. Here, Ayurveda is now much sought after. Twenty years ago, relatively few people in Europe, apart from those whose origins lay in South Asian countries, knew about Ayurveda; 5 years ago Ayurveda played a leading role within a trend of deeply relaxing wellness therapies. Today Ayurveda is increasingly acknowledged to be a comprehensive, traditional system of holistic medicine and has reached the threshold of entering into the realm of evidence-based science.

In several European countries, such as Italy and the UK in addition to Germany, doctors can now study Ayurvedic medicine within the framework of postgraduate medical education recognized by medical councils[1] and universities.[2,3] To increase interest in Ayurvedic medicine, massage, nutrition, and therapy, a multitude of seminars and training programs are now offered. Ayurveda has also gained ground clinically in Europe: besides Ayurveda treatment centers and hospitals, prestigious clinical projects have started officially recognizing Ayurveda’s value as a complementary medical system.

### The Rosenberg European Academy of Ayurveda

The Rosenberg European Academy of Ayurveda (REAA, www.ayurveda-academy.org) was founded in 1993. It is a nonprofit organization mainly financed by students’ tuitions and patients’ treatment fees, as well as by a relatively small amount of tax-deductible donations. Therefore, the challenge for the REAA in an otherwise highly subsidized health and education sector is quite high. As a nonprofit organization for holistic health and education, REAA pursues social purposes rather than economic ones. For the last years, REAA has played the role of a spearhead in pioneer work for the recognition and establishment of Ayurveda in German-speaking countries.

In addition to its Ayurveda Health and Treatment Centre in Birstein, Germany, REAA runs training centers in Austria and Switzerland, as well as other locations in Germany. For the past 15 years, it has conducted courses on Ayurveda massage, nutrition, and Ayurvedic medicine, training over 3000 adult students in long-term programs. Some 13,000 adult students, including 500 medical doctors and 2000 medical professionals, have attended its further educational programs on various aspects of Ayurveda and Yoga. In such ways, REAA has played a significant role in the development of Ayurveda in Europe.

### REAA Organization

REAA is organized in five departments:

1. The Department of Ayurvedic Medicine, which provides postgraduate or CME trainings for medical professionals,
2. The Department of Ayurvedic Nutrition, Massage, and Therapy,
3. The Department of Yoga, Vedic Psychology, and Sciences,
4. The Department of Ayurvedic Wellness, Business, and Management, and
5. The Ayurveda Health and Treatment Centre.

These departments work hand in hand to develop REAA’s Ayurvedic treatment and education programs. They are supervised by the REAA Academic Advisory Board and the Quality Assurance Agency for Higher Education (QAA) and other quality-assurance institutions.

In addition to a Department Head, the Department of Ayurvedic Medicine and the Ayurvedic Health and Treatment Centre are both supervised by a Medical Director (Dr. Ludwig Kronpass, Chief Physician of Gynaecology, public hospital, Bavaria) and monitored by the local health authorities.

### REAA teaching staff

REAA’s lecturers and teaching staff, who deliver the program, include visiting professors from universities such a GAU and BHU, and teachers from Kerala, Bangalore, and Coimbatore in South India who conduct about 50% of the lectures and workshops. Teaching staff also include medical professionals from German-speaking Europe, who specialize in Ayurvedic medicine, and have a long-standing experience with clinical Ayurveda in the EU. For example, three of our faculty have written the majority of high-standard Ayurveda publications in German.[4,9]

### Holism in health and education

REAA aims at enabling its students to restore and protect their client’s health and improve their quality of life, and so provide sustainable solutions to their health problems based on Ayurvedic medicine.

1. *Higher education*. A link to Middlesex University, London, provides students with the opportunity to gain a Master of Science, the first qualification in Ayurvedic Medicine, from a European university. The Professor of Clinical Natural Medicine at Charité Medical University, Berlin, advises our students on their dissertations, greatly increasing the value of their academic degree.
2. High academic quality is ensured by international partnerships in research and education and by constantly updating curricula.
3. Successful training requires outstanding staff and program leaders at its heart. All our teachers are selected for sound knowledge and long-standing experience in their subject matter and for high levels of competence in Ayurveda.
4. Individual support and supervision and transfer of professional knowledge using integrative teaching methods, supervisions, and practical training, and regular assessments, improve the quality of knowledge.
5. Practical training in Ayurvedic treatment programs is provided by placement at the treatment center in Birstein or at one of our partner hospitals in India.

### International relations

Close relations have been established with leading Indian and European universities and institutions such as Middlesex University in London (www.mdx.ac.uk/courses/postgraduate/complementary_health/ayurvedic_med_msc.aspx), and Charité Medical University in Berlin (www.charite.de/epidemiologie/english/pkomplement.html), Central Council for Research in Ayurveda and Siddha (CCRAS), Banaras Hindu University (BHU), Gujarat Ayurved University (GAU), Foundation for Revitalisation of Local Health Traditions (FRLHT), AVT Institute for Advanced Research, Coimbatore and the Mahagujarat Medical Society with its Ayurveda College in Nadiad, most of which have been formalized by MoUs. These enable the REAA to improve quality by using traditional Ayurvedic expertise. Many Ayurvedic professors, doctors, and practitioners as well as Indian political officials have taken the opportunity to visit the academy, either as members of the teaching staff, as speakers at congresses or for the purpose of ensuring the quality of the academy’s work. As a result, REAA’s Academic Advisory Board includes professors from Indian and European universities and Ayurveda doctors, vaidyas, and therapists who have taught its courses. As one of REAA’s main bodies the Academic Advisory Board was established to advise the directors of the departments and managing directors in all didactic and scientific matters. The members of the Academic Advisory Board exchange thoughts about fundamental and current problems to do with training and further education in Ayurveda. The Academic Advisory Board also determines the rules for examinations conducted at the academy and acts as a controlling authority.

Many of the currently leading training concepts of REAA have played a pioneering role in the development of professional Ayurvedic education in Europe (such as the training as Holistic Ayurveda Nutritionist or Psychological Ayurveda Consultant), and provided Ayurveda’s response to the public demand for holistic and natural health care. College lecturers from our MoU partner universities, reputed Ayurveda doctors, and experienced therapists have helped in designing the educational programs, to which they have contributed broad knowledge and experience. The programs have been developed in strict accordance with the guidelines of the Quality Assurance Agency for Higher Education, UK (QAA), making high didactic and academic quality a universal feature. In 2009, a research collaboration with the Institute of Social Medicine, Epidemiology and Health Economics at Charité Medical University, Berlin, was initiated to actively promote scientific investigation of Ayurvedic Medicine in Europe. All these make REAA’s broad offer of specialized training and further educational seminars unique in their diversity and qualifications offered. Students now come from countries all over the continent to the REAA to study Vedic sciences.

Therefore, the REAA has developed a Europe-wide perspective. For example, it has been connected with several Ayurvedic associations in Europe (European Professional Association of Ayurvedic Practitioners and Therapists, in German, the Verband Europaeischer Ayurveda-Mediziner und Therapeuten (VEAT); Swiss Professional Association of Ayurvedic Practitioners and Therapists (VSAMT); and the International Ayurveda Foundation (IAF)) which form the largest professional Ayurveda associations in Europe. REAA is committed to quality assurance of complementary and alternative medicine within the European Society for Quality in Healthcare, Vienna (ESQH). Consequently, the director was recently invited by the Department of AYUSH, Ministry of Health and Family Welfare of the Government of India, to be Chairman of the International Working Group for Ayurveda Education. He particularly advocates recognition and quality assurance of Ayurveda as well as networking in Europe.

### The Masters program (MSc in Ayurvedic medicine)

In its 4-year part-time course, “Master of Science in Ayurvedic Medicine,” the REAA offers a comprehensive further education in Ayurvedic Medicine in German-speaking Europe. The intensive 4-year training program is specially designed for medical doctors. Its 3150 h include almost 1000 h of practical clinical training. It leads to an official European “Master of Science” (MSc) degree valid throughout Europe since it is validated by the UK’s Middlesex University, London, with 240 UK credits equivalent to a minimum of 90 ECTS.

The final MSc module (of 9) is supervised by the Charité Medical University, Berlin, one of the highest ranked medical universities in Germany, and one of Europe’s leading CAM faculties. This guarantees that master’s dissertations meet the highest academic and scientific standards. It represents a broader accreditation within the scientific medical community. The program, structure and educational content has contributed decisively, besides the ones designed by the College of Ayurveda, Middlesex University, UK, and Ayurvedic Point, Italy, to the recently drawn-up standard European educational program in Ayurvedic medicine for medical doctors. It was originally proposed to the Department of Ayurveda, Yoga, Unani, Siddha and Homeopathy (AYUSH), Ministry of Health and Family Welfare, Government of India, and is now subject to evaluation by it.

Numbers show demand to have been stable and relatively high for several years. As of March 2010, 126 students are registered with REAA’s Department of Ayurvedic Medicine. Over 200 qualified practitioners of Western or natural medicine have already participated in the long-term courses on Ayurvedic Medicine. This high interest is also reflected in recent press coverage, with articles in major newspapers, magazines, and scientific journals.

On a more personal, extra-curricular note, the REAA hopes to help its students find new perspectives on their work. Healthy surroundings, friendly interactions with other participants, and a supportive spiritual program can create new perspectives on everyday life making it healthier and more self-fulfilling - a way to full inner peace. We hope that each stay will provide new strength and motivation on a student’s path so that he or she discovers and realizes a genuine vision in their life. With this, REAA’s Ayurvedic education, without entirely forfeiting its classical form, is made practical and suitable for western students. Having an MSc qualification in Ayurvedic medicine provides the student with the unique possibility of responding to the growing western patients’ demand for natural therapeutics and individual diagnosis and treatment. As a consequence, there is improved access to patients due to a completely new and holistic understanding. The Ayurveda training program opens up valuable new perspectives on health and disease and serves as a basis for holistically oriented medical practice in OPD, IPD, and prevention contexts. Students coming to the REAA are primarily searching for successful approaches to medicine and therapy, especially in the area of chronic disorders, and those related to the psyche. These they find in the science of Ayurveda.

### First comprehensive European research project on Ayurvedic medicine

A characteristic feature of Ayurveda is the importance laid on insights of ancient scholars found in the traditional texts, which good Ayurveda teachers and practitioners should constantly explore in their daily practice. In Ayurveda, research is not limited to current new developments. As regards academic and scientific research, however, Ayurveda is still in a pioneering phase in the West, and, apart from some isolated cases, little systematic research has been conducted on it so far.

To help remedy this situation, the REAA approached Professor Andreas Michalsen from the Institute of Social Medicine, Epidemiology and Health Economics at Charité Medical University, Berlin, in 2009. It is now participating in a collaborative research project: “International controlled and multi-centered study on the effectiveness of Ayurvedic Medicine in the treatment of osteoarthritis of the knee,” both as a research partner of Charité Medical University, Berlin, and the Central Council for Research in Ayurveda (CCRAS) of the Indian Ministry of Health.

The trial, designed as a 4-year study, is the first comprehensive study on Ayurvedic medicine in Europe since the turn of the millennium. The therapeutic part of the trial is supported and guided by the Rosenberg Society. An additional tri-partner agreement between CCRAS, Charité Medical University, and REAA, stipulating the frame conditions for a joint realization of a comparative study states that CCRAS will conduct the trial in India in addition to the one conducted in Germany using the same protocols. Outcomes of the study will be integrated with the German project.

### Ayurveda health and treatment centre in Birstein

As an affiliate of its academy, the REAA maintains its own Ayurveda Health and Treatment Centre. Patients can experience Ayurvedic medical treatment (including *Pancakarma*) and other guests restore their inner balance by Ayurvedic therapy. Far from everyday life and in the midst of nature, they can find inner peace, relaxation, and restored health. Ayurveda doctors, practitioners of natural medicine, and therapists from India and Germany treat our patients in groups of not more than 14 people. Visiting Professor Gupta supervises all *Pancakarma* treatments. Over the years, more than 1500 patients and guests have undergone traditional individualized Ayurveda treatments and health-promoting therapies, in a regime that includes excellent Ayurvedic cuisine, Yoga sessions, lectures, meditation, and workshops on disease prevention and health promotion.

### Symposium – Professional exchange – Encounter

The REAA’s annual International Ayurveda Symposium has been among Europe’s most valued Ayurveda conferences for many years. Lectures, speeches, and panel discussions are presented by internationally known professors, doctors, and specialists of Ayurveda from South Asia and various European countries. It represents the best of symposia and other meetings hosted by REAA. The symposium journals[10] published by the REAA within the frame of this important Ayurvedic event in Europe contain expert articles by participating speakers and lecturers.

Within the frame of the symposium, the academy organizes an Ayurveda camp on healing and research. True to the motto *Ayurvedic Medicine for Everyone*, the REAA offers Ayurvedic medical consultations and advice free of any charge, under medical direction, and with experienced Ayurvedic doctors and Vaidyas.

### European Congress for Integrative Medicine, Berlin, 2010

The European Congress for Integrative Medicine (ECIM) “… offers an innovative platform for medical practitioners and healthcare professionals as well as scientists, sponsors, and health politicians. The aim is to actively influence the development of the best possible health care. Besides international renowned experts in the field of Integrative Medicine medical practitioners from outpatient and inpatient settings are invited to participate in a lively exchange of ideas. Integrative medicine combines conventional Western medicine with complementary methods of treatment such as naturopathy, homoeopathy, and acupuncture. By overcoming the ideological divides between these systems, Integrative Medicine has the potential to provide the best possible medical care to each individual.”[11]

In cooperation with Charité Medical University, Berlin, the REAA organized a scientific Ayurveda forum to take place within the frame of this year’s 3rd European Congress for Integrative Medicine (ECIM) in Berlin on 3rd and 4th December, 2010. The Ayurvedic Forum, on the Congress’s second day provided the expert audience with four lectures on specific Ayurvedic topics served as an introduction and entry into a subsequent panel round,. It brought the possibilities and benefits of Ayurvedic medicine closer to the European medical professional circles and, therefore, enhanced its further establishment in European health politics.

### Quality in education – A global perspective as the major aim of the REAA

All these areas of operation serve to promote and establish Ayurveda in Germany as a profession appreciated by the population, and also on political and professional levels. REAA has managed to play a significant role in the growth of Ayurveda in Europe, especially in Germany, Switzerland, and Austria. The standard quality criteria and requirements the REAA adheres to in designing, organizing, and delivering educational programs form a major part in this respect, as it is the only way of getting courses and seminars accredited on professional levels. This is reflected by several quality seals and certificates awarded to REAA in the past years, such as the one according to the Regulations on the Recognition and Approval of Further Education and Training, in German the Anerkennungs- und Zulassungsverordnung Weiterbildung (AZWV). AZWV certification includes all major parts of ISO9001, and was especially developed for the recognition and approval of high-quality schools and institutions providing adult education.

The REAA is highly committed to keeping its educational mission at a high standard. In this context, it is involved in the Global Propagation of Ayurveda, initiated by the Department of Ayurveda, Yoga, Unani, Siddha, and Homeopathy (AYUSH), Government of India. Its Director was appointed Director of the International AYUSH Working Group on Ayurveda Education, consisting of 21 delegates from 9 countries. Its aim is to design and develop some qualified and global model curricula for education in Ayurveda, which are also accredited by the Central Council for Indian Medicine (CCIM) and the Department of AYUSH.

In this context, international guidelines and quality assurance in Ayurveda are necessary aspects for the promotion of Ayurveda in western countries. Quality needs to be maintained in Ayurvedic education, in order to provide good educational quality to students and ensure good practice of Ayurvedic medicine and therapies. This can only be achieved by upholding Ayurveda’s traditions and gaining practical experience in India. Teaching staff with real experience of Ayurvedic medicine and therapies in South Asia need to be involved in Ayurvedic education. Here the principle of Gurukula has to be mentioned, as it forms a very traditional and in-depth way of teaching the integrative knowledge of Ayurveda and molding future Ayurvedic doctors’ or therapists’ personality. Indian Universities also need to be involved since they teach Ayurveda as practiced in India today.

However, an important issue in designing a standard global curriculum for Ayurveda is the fact that different conditions and laws on education prevail in different countries. These have to be taken into account during the development process. South Asia’s Ayurveda needs to adapt to such conditions without losing its original character. Emphasis on holism and spirituality should be maintained, though the latter might be likely to be questioned in professional medical circles in Europe and all over the world. A way to weaken objections within these circles must be through science. Modern medicine used scientific and research to substantiate its therapies. Ayurveda needs to use it, too, if it wants to move forward. Only by integrating essential researches, trials, and surveys, can Ayurveda gain accreditation in other countries outside South Asia. That will lead to clear definitions for job descriptions for Ayurveda, something that is still lacking in many Western countries, including Germany.

Ayurveda should be globalized as a complete system; instead of getting accreditation as part of a modern integrated or complementary concept of medicine, it should be valued as a traditional medicine, that is surely open to modernization, but likes to adhere to its classical literature, as this forms the real basis for all Ayurveda teaching and practice. Students and practitioners of Ayurveda abroad, however, focus on other aspects as regards the propagation of Ayurveda and its education. They do not like to learn things that they cannot practice. They wish to see examples of Ayurveda in practice, to prepare for personal interactions with clients and patients in their own country. However, most students are busy with their profession and life and being unable to spend enough time in India to gain practical experience, are torn between work obligations and their desire to gain practical experience in Ayurveda. In this context, Sanskrit should be mentioned - the classical language of the ancient Ayurvedic texts is compulsory in Ayurvedic education. Students and practitioners may be eager to understand Ayurveda’s classical concepts but do not see the necessity to acquire complete knowledge of the Sanskrit language in order to understand them. Their ambition is to explore the intelligence of the Ayurvedic system and so become able to help people in a holistic way and to integrate their understanding with other therapy concepts. Regaining the true ideal of their profession as doctor constitutes a major motivation to study Ayurveda after modern medical education or training as a therapist has been completed.

The above have been carefully considered in developing and refining REAA curricula. They satisfy the need to maintain respect for Ayurveda’s traditional aspects, as well as western students’ needs for practical knowledge and innovative teaching techniques. They should be carefully considered within the AYUSH Working Group on Education in Ayurveda, or any other institution aiming to develop high-quality curricula for further education in Ayurveda.
